# Predictive classification of Alzheimer’s disease using brain imaging and genetic data

**DOI:** 10.1038/s41598-022-06444-9

**Published:** 2022-02-14

**Authors:** Jinhua Sheng, Yu Xin, Qiao Zhang, Luyun Wang, Ze Yang, Jie Yin

**Affiliations:** 1 College of Computer Science, Hangzhou Dianzi University, Hangzhou, 310018 Zhejiang China; 2 Key Laboratory of Intelligent Image Analysis for Sensory and Cognitive Health, Ministry of Industry and Information Technology of China, Hangzhou, 310018 Zhejiang China; 3 Beijing Hospital, Beijing, 100730 China; 4 National Center of Gerontology, Beijing, 100730 China; 5 Institute of Geriatric Medicine, Chinese Academy of Medical Sciences, Beijing, 100730 China

**Keywords:** Cognitive ageing, Neurological disorders

## Abstract

For now, Alzheimer’s disease (AD) is incurable. But if it can be diagnosed early, the correct treatment can be used to delay the disease. Most of the existing research methods use single or multi-modal imaging features for prediction, relatively few studies combine brain imaging with genetic features for disease diagnosis. In order to accurately identify AD, healthy control (HC) and the two stages of mild cognitive impairment (MCI: early MCI, late MCI) combined with brain imaging and genetic characteristics, we proposed an integrated Fisher score and multi-modal multi-task feature selection research method. We learned first genetic features with Fisher score to perform dimensionality reduction in order to solve the problem of the large difference between the feature scales of genetic and brain imaging. Then we learned the potential related features of brain imaging and genetic data, and multiplied the selected features with the learned weight coefficients. Through the feature selection program, five imaging and five genetic features were selected to achieve an average classification accuracy of 98% for HC and AD, 82% for HC and EMCI, 86% for HC and LMCI, 80% for EMCI and LMCI, 88% for EMCI and AD, and 72% for LMCI and AD. Compared with only using imaging features, the classification accuracy has been improved to a certain extent, and a set of interrelated features of brain imaging phenotypes and genetic factors were selected.

## Introduction

Alzheimer's disease (AD) is a complicated neurodegenerative disease involving a variety of pathogenic factors (biological and psychosocial). As the condition worsens, patients often suffer from mental and cognitive disorders, memory decline and behavior changes, which affect people's normal life ability. Mild cognitive impairment (MCI) is a state between normal and dementia which can be considered the early stage of AD. Nearly 10–15% of MCI patients are converted into AD patients every year^1^. Except for a few number of familial cases driven by genetic mutations, the main pathogenic factors of AD are still unclear^2^. In 2017, AD has become the sixth leading cause of death in the United States^3^. According to the International Alzheimer’s Disease (ADI) report in 2019, approximately 95% of the public believe that they may suffer from AD in the future^4^. If the disease can be detected early and measures can be taken timely, the onset of AD can be effectively delayed^5^. Therefore, early diagnosis and early intervention are essential for the control of AD.

The increasing development of neuroimaging has brought new vitality to the study of human brain structure and function. Frequently-used brain imaging techniques include Magnetic Resonance Imaging (MRI)^6^, Diffusion Tensor Imaging (DTI)^5^, Positron Emission Tomography (PET)^7^. Many researches focused on how to use one of them or combine multiple imaging modalities to classify AD. Li et al.^8^ designed a powerful deep learning system to identify different stages of AD patients based on MRI and PET. Bi et al.^9^ proposed a random support vector machine clustering method to classify AD and HC, with an accuracy rate of 94.44%. At present, the accuracy of AD and HC using imaging materials can reach more than 90%, while the accuracy of HC and MCI is relatively low. There are two main reasons for low accuracy of early diagnosis. First, cerebral atrophy is a gradual process, which is relatively subtle and difficult to detect in the early stages. Second, there is a certain overlap in the data space between the normal aging of the brain of normal people with age and the brain atrophy of early MCI patients.

In recent years, the integration of brain imaging and genetic data for research has become an active research topic^10^. For genetically complex diseases, at the level of a single nucleotide polymorphism (SNP), it is impossible to determine the main cause of the difference. Brain imaging genomics conducts comprehensive analysis of brain imaging and genomic data to obtain new insights, which have bright prospect for a better understanding of disordered brain functions. Through high-throughput genotyping technology, Genome Wide Association Studies (GWAS)^11^ determined the high-density genetic marker SNPs or gene copy number variation of large-scale population DNA samples at the whole genome level. It is a strong way to identify disease susceptibility loci. Researchers used GWAS to analyze SNP data and found that genetic factors play a significant role in the development of AD^12,13^. Dukart et al.^14^ obtained an accuracy of 76% via Naive Bayes to identify converter and stable MCI with glucose positron emission tomography as a single biomarker. The accuracy increased to about 87% when including further imaging data and APOE information. Dukart’s experimental results indicated that adding genetic factors can indeed help image features to improve classification accuracy.

With the rapid development of machine learning and deep learning, people have found that it can be used as an auxiliary diagnostic method, such as SVM^15^ and convolutional neural network^16,17^. There are several difficulties in the joint study of brain imaging and genetic data for classification and prediction: (1) High-dimensional data can cause computational and statistical problems^18^, and different modalities are heterogeneous; (2) Models may encounter multicollinearity problems for potentially correlated high-dimensional genetic variables^19^; (3) High-dimensional genetic data contains a lot of redundant information^19^.

**Figure 1:**
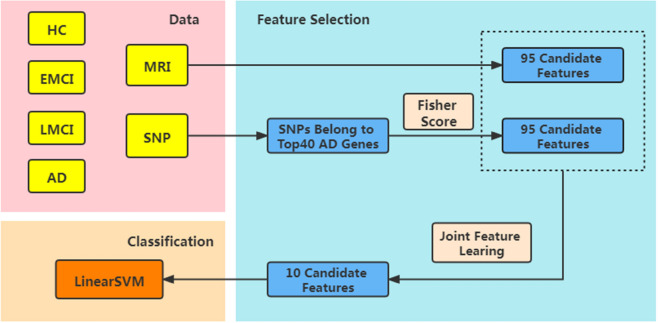
Specific steps of our method.

How to effectively study genetic information and image phenotypes, while fully considering the heterogeneity of data and the robustness of the model, is a major challenge in the application of image genetics. In order to improve the accuracy of AD diagnosis and make full use of the supplementary information between different modalities, we integrate Fisher score and multi-modal and multi-task feature selection to learn brain imaging and genetic data. Fisher score was used to pre-reduce high-dimensional genetic features and eliminated genetic features with small contributions. Genetic features obtained and brain imaging data were used for multi-task joint feature selection. Then, we used linear support vector machine (SVM) to predict healthy controls (HC), early MCI (EMCI), late MCI (LMCI) and AD patients. Finally, we systematically evaluated the potential of modal combinations and verified the effectiveness of the method. Figure 1 shows the steps of our method.

## Results

To avoid the possible impact of different image acquisition equipment and genotyping techniques, data used in this article were obtained from the Alzheimer’s Disease Neuroimaging Initiative (ADNI) database stage 2. Considering the category balance problem, we screened 100 subjects with brain imaging and genetic data measured at the same age. The study sample (N = 100) included 25 HC subjects, 25 early MCI, 25 late MCI and 25 AD subjects. The average age is 73.597 years, and the male to female ratio is 60:40. They have quality-controlled quantitative brain imaging data and genetic data. The demographic and clinical characteristics of participants, summarized by the diagnosis, are shown in Table 1 below.

**Table 1:** Demographic characteristics of subjects.

Diagnostic	Male/Female	Age (mean[min–max])	Education
Healthy control	15/10	73.44 [65.1–84.9]	17.12
Early mild cognitive impairment	14/11	71.04 [61.9–82.3]	16.04
Late mild cognitive impairment	15/10	73.47 [55.0–91.4]	16.64
Alzheimer’s disease	16/9	76.44 [55.9–90.3]	15.80

### Classification performance

In the experiment, we evaluated the performance of the method in different cognitive groups: (1) HC and EMCI, (2) HC and LMCI, (3) HC and AD, (4) EMCI and LMCI, (5) EMCI and AD, and (6) LMCI and AD. Due to the limited number of subjects, we used fivefold cross-validation (CV) to evaluate model performance^20^. In fivefold CV, we randomly divided the data set into 5 parts, with 1 part for testing and the remaining part was used for training. Repeat this process 5 times so that each part was tested once. In order to obtain a more reliable performance estimate, we calculated the average of the test accuracy of 5 tasks as the evaluation standard. Table 2 lists the cross-validation accuracy when using different machine learning methods for group recognition. We can see that SVM is the relatively most suitable classification algorithm.

**Table 2:** Cross validation accuracy in identification of groups using different machine learning methods.

	HC vs EMCI (%)	HC vs LMCI (%)	HC vs AD (%)	EMCI vs LMCI (%)	EMCI vs AD (%)	LMCI vs AD (%)
SVM	82	86	98	80	88	72
KNN	80	86	96	76	82	72
Tree	70	70	92	76	88	74
Ensemble	72	72	94	66	86	74

We considered the classification performance of three different input biomarker combinations based on linear SVM. The three morphological data tested were: (1) SNP, (2) sMRI, (3) sMRI and SNP. Table 3 shows the classification performance obtained by three different input biomarkers.

**Table 3:** Classification performance comparison of different modes.

	HC vs EMCI (%)	HC vs LMCI (%)	HC vs AD (%)	EMCI vs LMCI (%)	EMCI vs AD (%)	LMCI vs AD (%)
SNP	50	50	58	52	46	40
sMRI	82	82	98	74	90	70
sMRI + SNP	82	86	98	80	88	72

We analyzed the performance of a single mode and compared it with the performance of a multi-mode. We can see that the performance of sMRI is far better than SNP, because brain structure changes are a phenotypic feature closely related to diagnostic labels. However, including both cerebrum and genetic features as model predictors enhanced the performance compared with using either cerebrum or SNP features alone. In most tasks, especially MCI recognition, the performance of genetic imaging data was better than that of a single modality. For example, the accuracy of using SNP+sMRI in HC and LMCI classification was 4% higher than that of sMRI alone; the accuracy of EMCI and LMCI was improved by 6%; the accuracy of LMCI and ADI was improved by 2%. However, for tasks such as HC and AD, EMCI and AD, which are relatively simple and have great differences in themselves, compared with the performance of a single image modal, the performance of multi-modality has not improved, and in some cases it may cause performance degradation. The main reason is that in the absence of SNP, the performance of the model has reached a saturated state. At this time, adding SNP data will be regarded as noise, which will have a negative impact on the performance of the classifier. Another reason is that the sample set we use is relatively small.

Studying the phenotype or SNP feature of each brain region separately will discard the potential correlation between the intra-modal features as well as between the features of different modalities. Univariate analysis can quickly provide important information between genetic features or imaging features and diseases. In order to further study the benefits of genetic and image data fusion learning, we compared with traditional univariate feature selection without considering the potential correlation between modalities. LR-RFE^21^ iteratively eliminates the features with the lowest contribution. It has been used and is expected to detect AD early and predict the progress of AD^21^. KPCA^22^ believes that the greater the variance of data distributed along a certain feature, the more information the feature contains. We used LR-RFE and KPCA to perform feature selection on image and genetic information respectively. Five of each imaging gene features were selected and applied to linear SVM for diagnosis.

**Figure 2:**
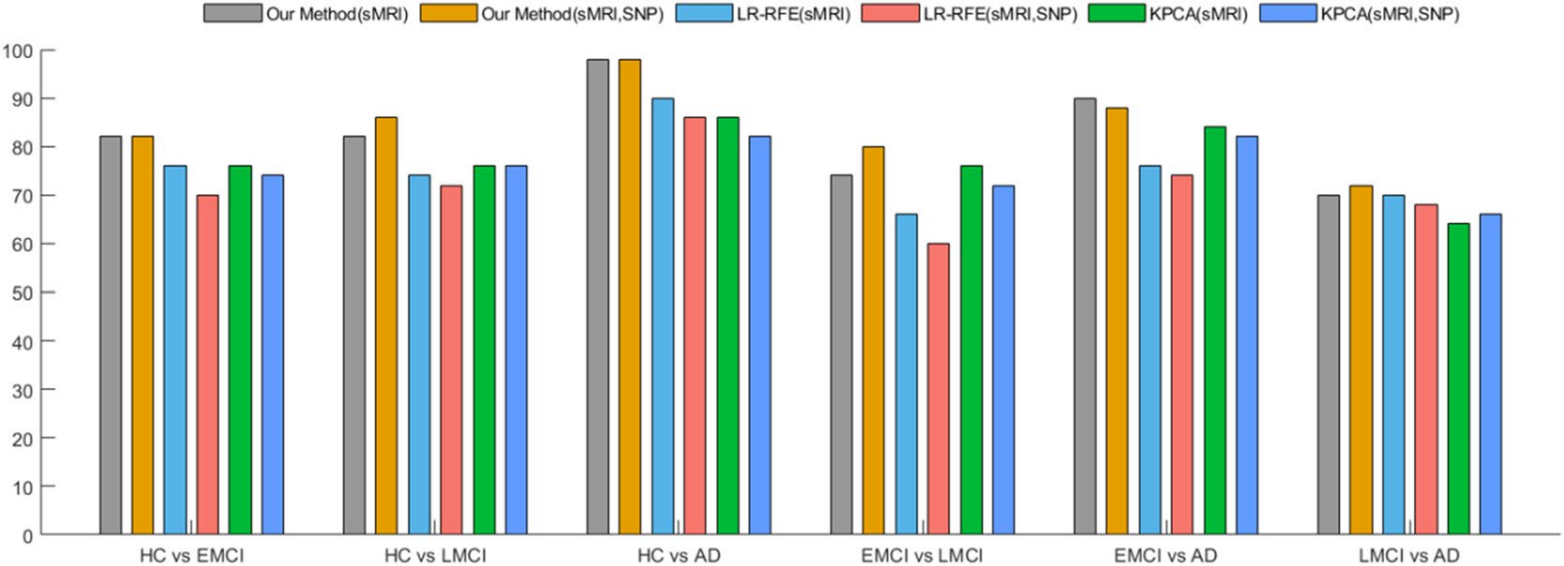
Classification performance of different feature selection methods.

Based on the performance of image features, we show the impact of three methods on classification performance after adding genetic features. Figure 2 below shows that the accuracy of traditional feature selection for those two modalities has decreased in most classification tasks. It illustrates that overfitting or increased noise may occur after adding SNP features. But our method has achieved good performance in all six binary tasks, and the performance has been further improved after combining SNP data. Different from traditional univariate feature selection, we studied the correlation between image and genetic data in a public space, considered the sparsity between different modalities through G_1_-norm, and used l_2,1_-norm regularization to jointly select genetic information related to important image data. In the learning process, the differences of different morphological features are fully considered, and different weights were assigned to each feature, which reduces the risk of overfitting the training data by the classifier. The results show that the fusion of genetic information can indeed take advantage of the complementarity between the modalities and eliminate the redundancy between the modalities, thereby obtaining better feature representation and improving classification performance.

### Selection of biomarkers

**Figure 3:**
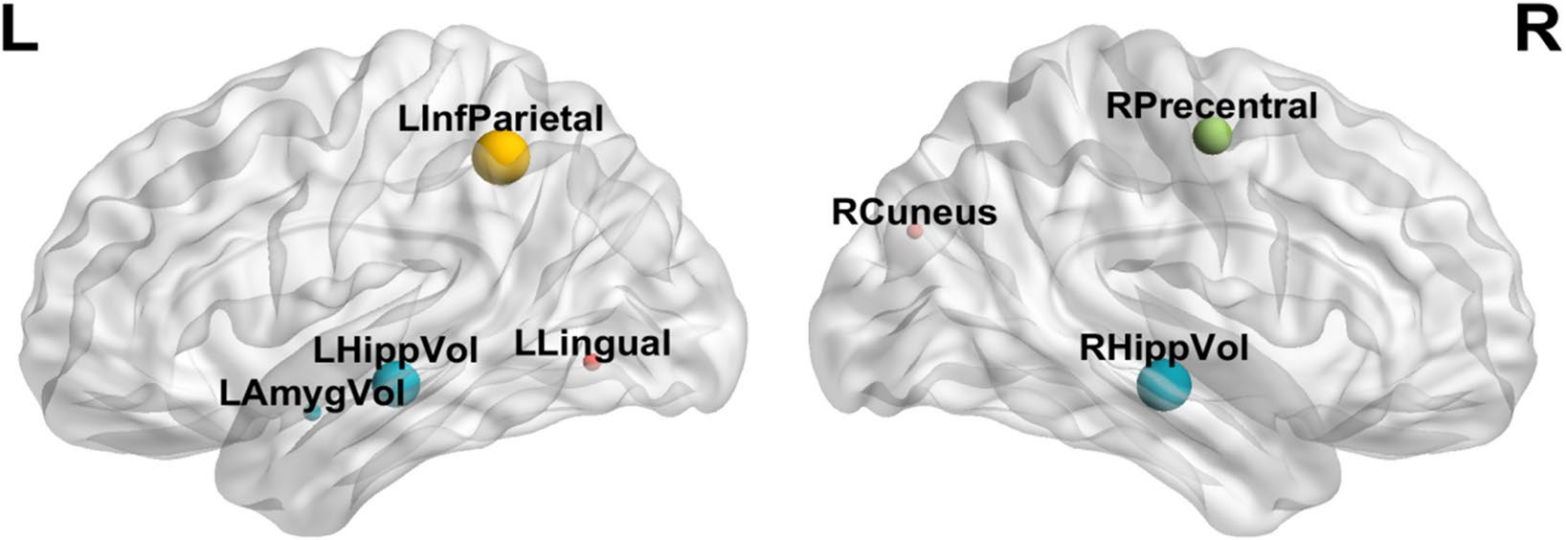
Brain distribution in the core brain area.

Finding out the most discriminating brain regions and SNPs is essential for the diagnosis of AD. The brain regions and SNP data selected most in the experiment can be used as potential biomarkers for clinical diagnosis. In Table 4, we give the brain imaging features that are selected every time in fivefold CV, and Fig. 3 shows the distribution of these brain regions in the brain. “HippVol” (hippocampal volume) plays the significant role in predicting memory performance. “LHippVol” serves as an important potential biomarker in the recognition of HC in the three stages of disease, implicating that it is an important indicator for cognitive decline and has a potential for early detection of AD. “LAmygVol” (amygdala volume) was also selected in the early diagnosis of HC and EMCI. This is because the hippocampus and amygdala are the first to form plaques during the development of AD^23^. Besides, “Precentral” (thickness of precentral), “Lingual” (thickness of lingual), “Cuneus” (thickness of cuneus) and “InfParietal” (thickness of inferior parietal) are also selected in other classification groups. Zhang et al.^24^ used an intrinsic brain-based CAD system to detect 30 brain regions related to AD, which was consistent with our results.

**Table 4:** Most selected sMRI features for diagnosis.

	HC vs EMCI	HC vs LMCI	HC vs AD	EMCI vs LMCI	EMCI vs AD	LMCI vs AD
ROI	LHippVol LAmygVol RPrecentral	LHippVol LLingual	LHippVol RHippVol RCuneus LInfParietal	RPrecentral	RPrecentral	LInfParietal

In terms of genetic information, the SNPs that have been selected many times for AD and HC classification come from the AOPE gene. APOE is related to neuroimaging measurement of diseases, especially the left hippocampus and right hippocampus^25^, which are the most significant risk factors for AD. In the diagnosis of MCI, the SNPs that are selected multiple times come from the CR1 gene and the SORCS1 gene. They are also well-known candidate genes related to MCI. CR1 mainly affects the development of AD by affecting Aβ deposition, brain structure and glucose metabolism during the progression of AD^26^. During the experiment, many SNPs were selected from the same gene, such as SORCS1 gene, CR1 gene and DAPK1 gene. In general, our research results are consistent with existing research, and provide help for the clinical diagnosis of AD and further exploration of AD treatment strategies.

## Discussion

Previous studies mostly used imaging features for disease prediction. Some researchers added APOE genes on the basis of imaging to improve performance. For example, Filipovych et al.^27^ proposed a method to predict the conversion of MCI to AD by compounding multiple imaging scores. They used the non-linear mode and the linear mode to obtain the subjects' imaging and genetic scores, and then synthesized the imaging genetic scores as the weighted sum of the imaging scores and genetic scores. The accuracy of imaging markers only is AUC = 0.746, and the accuracy is improved to AUC = 0.779 after including imaging genetic markers. However, Filipovych's experiment did not consider the correlation between imaging and genetic characteristics, and simply combined them through scoring. In Table 5, we gave a performance comparison mainly for recent studies achieving classification accuracy, sensitivity and specificity.

**Table 5:** Example studies for outcome prediction via integrating imaging and genomics data.

Sr. no	Year	Authors	Modality	Dataset	Method	Target	Performance		
							Acc (%)	Sens (%)	Spec (%)
1	2016	Dukart et al. ^12^	FDG-PET, AV45-PET, sMRI, APOE	708(144AD, 265sMCI, 177cMCI, 122HC)	Bayesian-Markov-Blanket + Naive Bayes	sMCI vs cMCI	86.8	87.5	86.1
2	2016	Peng et al. ^28^	MRI, PET, SNP	189(49AD, 93MCI, 47NC)	Krenel-learning	AD vs NC	96.1	97.3	94.9
						MCI vs NC	80.3	85.6	69.8
3	2017	Singanamalli et al. ^29^	MRI, CSF, FDG-PET, APOE, cognitive measures	149(52AD, 71MCI, 26HC)	Cascaded multi-view canonical correlation (CaMCCo)	CN	89	59	96
						MCI	80	88	80
						AD	80	69	88
4	2017	Liu et al. ^30^	sMRI, APOE, FDG-PET, cognitive measures, demographics	426(121AD, 126MCI-c, 108MCI-nc, 180NC)	ICA + Cox model	MCI-c vs MCI-nc	84.6	86.5	82.4
5	2018	Ning et al. ^31^	MRI, SNP	721(138AD, 358MCI, 225CN)	Neural network	Conversion from MCI to AD	–	–	–
6	2019	Zhou et al. ^32^	MRI, PET, SNP	347(101AD, 138MCI, 108NC)	Neural network	NC vs MCI vs AD	–	–	–
						NC vs sMCI vs pMCI vs AD	–	–	–
						NC vs MCI	–	–	–
						NC vs AD	–	–	–
7	2019	Spasov et al. ^33^	sMRI, APOE, cognitive measures, demographics	785(192AD, 181pMCI, 228sMCI, 184 HC)	Multi-tasking neural network	sMCI vs pMCI	86	87.5	85
8	2020	Brand et al. ^34^	sMRI, SNP	723(170AD, 352MCI, 201HC)	Task balanced multimodal feature selection	AD vs HC/MCI	72.8	–	–
9	2020	Bi et al. ^35^	fMRI, SNP	109(37AD, 37EMCI, 35HC)	Cluster evolutionary random forest (CERF) + SVM	AD vs HC	81	–	–
						EMCI vs HC	80	–	–
10	2021	Sheng et al. (this paper)	sMRI, SNP	100(25AD, 25LMCI, 25EMCI, 25HC)	Fisher score + Multi-task feature selection + SVM	AD vs HC	98	100	96
						AD vs EMCI	88	88	88
						AD vs LMCI	72	72	72
						LMCI vs HC	86	88	84
						LMCI vs EMCI	80	88	72
						EMCI vs HC	82	80	84

Although we have achieved good results in six traditional binary classification tasks, there are still some limiting factors. To start with, in order to determine a group of subjects that have both the image and heredity measured at the same time and fully consider the category balance, we have to discard a large amount of available data in the ADNI database, resulting in a very limited sample size for the training and testing data sets. Second, we only used two modes to calculate features. In fact, in addition to MRI and SNP data, ANDI has many other forms of characteristics, such as PET, demographics, and neuropsychological assessments. These patterns may carry important pathological information or provide supplementary information between modalities. Third, since the score of each feature of the Fisher Score is calculated independently, the selected feature is sub-optimal, and it is not possible to select features with relatively low individual scores but high scores when they are combined. Finally, because brain atrophy is a gradual process, it is relatively subtle and difficult to detect in the early stages. In the experiment, we did not fully consider the normal shrinkage of some people with age.

We will use new Freesurfer versions with more accurate anatomical measurements in our future work. In our future work, we are also committed to the following research: (1). plan to obtain more subjects with more varied patterns of data, and explore the impact of identify more characteristic correlations between patterns on disease diagnosis brain imaging and genetic markers with disease development, (2). in order to fully consider age factor and reduce the impact of normal aging on classification performance, we need to add a reasonable age-related regression model to the optimization formula to reduce impact to the model from normal aging, (3). we need to re-improve the dimensionality reduction method of genetic features to give play to genetic information in AD diagnosis, and (4). more challenging and clinically diagnostic multi-classification tasks.

## Conclusion

There is a certain overlap in the data space between early brain atrophy of patients and normal aging of the brain of healthy people, which leads to low accuracy of many computer-aided diagnosis methods. In this article, both image and genetic features are considered as candidate features for classification. By effectively integrating consistent brain imaging and genetic features through methods such as pre-dimensionality reduction and feature selection, patients with EMCI, LMCI and AD can be more accurately identified from HC. A set of characteristics related to imaging phenotypes and genetic factors were selected, and the selected risk characteristics were basically consistent with existing research. We selected 5 brain imaging and 5 genetic features for disease process diagnosis through the feature selection program, and achieved good classification accuracy. Although the SNP feature has a weak predictive ability for the development of AD, it can help the imaging mode to improve performance together.

## Material and methods

Data collection and sharing for this project was funded by the Alzheimer’s Disease Neuroimaging Initiative (ADNI) (http://adni.loni.usc.edu). Informed consent was obtained from the volunteer in accordance with the institutional review board policy. All methods were carried out in accordance with relevant guidelines and regulations. All experimental protocols were approved by the institutional review board (IRB) at Hangzhou Dianzi University (IRB-2020001).

### Data preprocessing

FreeSurfer is suite of tools that provide extensive and automated analysis of cerebrum region^36^. It can conveniently process brain MRI images, and generate high-precision gray and white matter segmentation planes and gray matter and cerebrospinal fluid segmentation planes. Based on these two surfaces, the thickness of the cortex at any position and other surface data characteristics such as cortical outer surface area, curvature, Gray matter volume, etc., these parameters can be mapped to the surface of the cerebral cortex obtained by the white matter expansion algorithm for visual display. FreeSurfer version 5.3 was used to extract 66 cortical thickness measurements and 29 volume measurements for each baseline MRI scan. Those measurements were pre-adjusted to eliminate the effects of the baseline age, gender, handedness, education, and intracranial volume (ICV). We used the above 95 regions of interest in the experiment.

The genotyping data were genotyped by the Human 610-Quad BeadChip and preprocessed according to standard quality control and imputation procedures. The value of SNP is 0, 1, or 2, which indicates the number of minor alleles. Most of the SNPs may have nothing to do with the pathogenesis of AD, and only a small part of them are high risk factors for AD and are related to changes in certain brain regions. We only used SNP data belonging to the top 40 AD candidate genes listed in the AlzGene database (www.alzgene.org) to screen out 916 SNP features. We finally obtained 95 + 916 = 1011 candidate features, which come from the two modalities of each subject.

There are differences in the size of each person’s brain. MaxMin-normalization related to extreme values. Unstable data sets and extreme maximum/minimum values may lead to data congestion after scaling. Based on this consideration, we adopted two different normalization approaches. We standardized the MRI data according to formula (1), and normalized the SNP data according to formula (2).

1 $$\tilde{x} = \frac{{x - x_{\min } }}{{x_{\max } - x_{\min } + \varepsilon }}$$

2 $$\tilde{x} = \frac{{x - \overline{x}}}{{\sqrt {\frac{1}{N - 1}\sum\nolimits_{i = 1}^{N} {\left( {x_{i} - \overline{x}} \right)}^{2} + \varepsilon } }}$$

where $\overline{x} = \frac{{1}}{N}\sum\nolimits_{i = 1}^{N} x_{i}$ *ε* is a very small positive number to avoid situations where the denominator approaches zero.

### Preprocessing of genetic data

The dimensionality of SNP features is generally high and most gene variants account for less than 1% of the measurement variance, so our genetic data is a high-dimensional sparse matrix. This is fatal for many machine learning models, especially models with gradient descent as the optimization algorithm. If it is directly used for joint learning with image data, a large amount of irrelevant genetic information may have a negative impact on the final selected feature subset. Therefore, before using genetic data for joint feature learning, we need to perform simple pre-dimensional reduction processing on genetic data to reduce the dimensionality to a level similar to image features. Feature selection can be divided into three types: filtering, wrapping and embedded^37^. In this paper, a filtering feature selection method independent of the classifier was used. This type of method usually selects a subset of features that are highly related to the category. Filtered feature selection methods are often used in the preprocessing of original data, which can better filter non-critical features, retain the main structural features with high correlation as much as possible, and finally reduce the dimensionality of feature set attributes.

The value and distribution of data do not want to change here, so that methods such as PCA and LDA are abandoned. After comparing the performance of Fisher score, mutual information, F-tests and minimum redundancy maximum relevance, we chose Fisher score. Fisher score^38^ is an effective feature selection criterion, which has the advantages of simple calculation, time saving, and high accuracy. Its main method is to find a subset of features according to Fisher’s linear discriminant, so that the selected features are different in the data space. The distance between data points of a class is as large as possible, and the distance between data points in the same class is as small as possible. Given a data set of N samples containing c classes, define the inter-class divergence S_b_ (x_i_) of the ith feature and the intra-class divergence S_t_ (x_i_) of the ith feature of the kth sample as

3 $$S_{b} \left( {x_{i} } \right) = \sum\nolimits_{k = 1}^{c} {n_{k} \left( {\mu_{i}^{k} - \mu_{i} } \right)^{2} }$$

4 $$S_{t} \left( {x_{i} } \right) = \sum\nolimits_{j = 1}^{{n_{k} }} {\left( {x_{ij}^{k} - \mu_{i}^{k} } \right)^{2} }$$

where *n*_*k*_ is the number of samples of class k, *µ*_*i*_ is the mean value of the ith feature of the whole sample, and $x_{ij}^{k}$ is the mean value of the ith feature of the jth sample in the k-class sample. When the inter-class divergence is as large as possible, the intra-class divergence is as small as possible, and the Fisher score of the ith feature can be expressed as follows

5 $$F\left( {x_{i} } \right) = \frac{{S_{b} \left( {x_{i} } \right)}}{{\sum\nolimits_{k = 1}^{c} {n_{k} S_{t}^{k} \left( {x_{i} } \right)} }} = \frac{{\sum\nolimits_{k = 1}^{c} {n_{k} \left( {\mu_{i}^{k} - \mu_{i} } \right)}^{2} }}{{\sum\nolimits_{k = 1}^{c} {n_{k} } \sum\nolimits_{j = 1}^{{n_{k} }} {\left( {x_{ij}^{k} - \mu_{i}^{k} } \right)^{2} } }}$$

The greater the Fisher Score value, the stronger the ability to distinguish features. After calculating the Fisher Score of each feature, we sort the scores in descending order and select the highest m genetic factors as the genetic input for the next step of learning.

### Multimodal joint feature selection

Two modes contain unique information and have a certain potential connection, both modes are expected to help the diagnosis of AD. Joint multimodal learning can help discover more powerful features than when learning alone. Multi-task learning is a sub-field of machine learning, which uses the commonalities and differences between different tasks to improve the generalization ability and prediction accuracy of the model^39,40^. When the square of the l_2_-norm is used as a loss function, it is insensitive to smaller outliers and sensitive to larger outliers, while the l_1_-norm as a loss function is just the opposite. Recently, many multi-task learning methods use group sparsity l_2,1_-norm to couple cross-task features together for joint feature selection^41^.

In the feature selection process, each category was as a separate task. Assuming that the data set X = [x_1_,x_2_,…,x_N_] ∈ R^d×N^ contains M modalities, the label set Y = [y_1_,y_2_,…,y_N_] ∈ R^c×N^, and the label adopts binary representation, that is, only one element in each row is 1, and the other elements is 0.

Through l_2,1_-norm, we can make the model better handle outliers and reduce the burden of tuning. Therefore, we used l_2,1_-norm to select features for multiple tasks. The expression of l_2,1_-norm is

6 $$\left\| W \right\|_{2,1} = \sum\limits_{i = 1}^{d} {\sqrt {\sum\limits_{j = 1}^{N} {w_{ij}^{2} } } } = \sum\limits_{i = 1}^{d} {\left\| {w_{i:} } \right\|_{2} }$$

where *w*_*ij*_ is the weight coefficient of the ith feature for category j.

**Figure 4:**
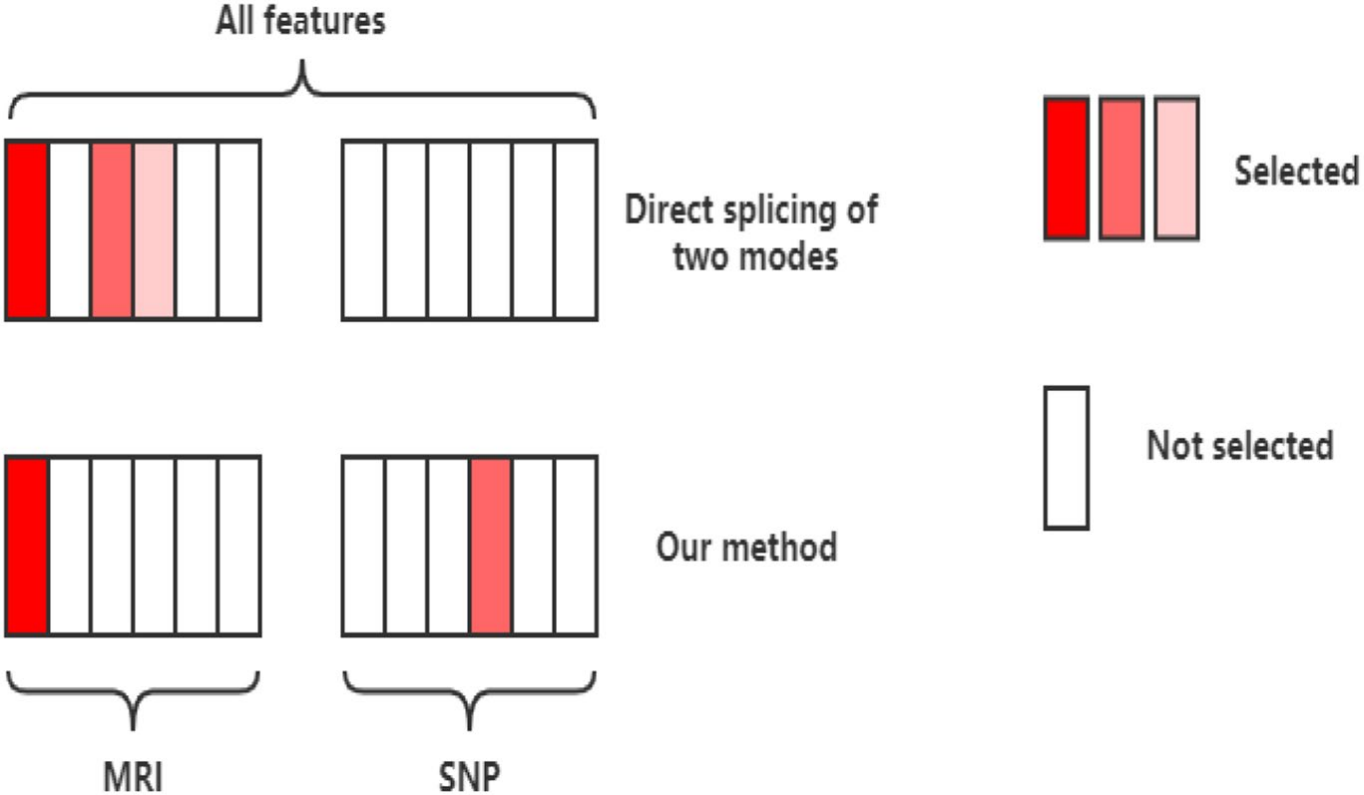
Feature selection diagram.

Because the characteristics of different modalities have different effects on the task, such as SNP data, their individual characteristics or overall characteristics are weaker than image characteristics. Compared with the intuitive changes in the volume of brain partitions, genetic data is more forward-looking. If genetic data and image data are directly combined for traditional feature selection, it is very likely that most or all of the selected features are image data, as shown in Fig. 4^28^. In the process of feature selection, if we do not impose proper constraints on our loss function, it may happen that even high-risk genetic features have generally low weights. However, l_2,1_-norm is an excessively strong group sparsity constraint, which may cause the modal to be discarded and ultimately affect the classification result. Wang et al.^42^ proposed a new group l_1_-norm (Group_1_-norm), which strengthened the sparsity between different modes by using l_2_-norm in each mode and using l_1_-norm between modes. Group1-norm is defined as

7 $$\left\| W \right\|_{{G_{{1}} }} = \sum\limits_{i = 1}^{c} {\sum\limits_{j = 1}^{M} {\left\| {w_{ij} } \right\|_{2} } }$$

Adding l_2,1_-norm and G_1_-norm to the loss function, we can finally express as the following form

8 $$\mathop {\min }\limits_{W} L\left( {X,Y,W} \right) + \gamma_{1} \left\| W \right\|_{{G_{1} }} + \gamma_{2} \left\| W \right\|_{2,1}$$

where *γ*_1_, *γ*_2_ > 0 is the regularization parameter. The relative importance of features is represented by the sum of absolute values of w. We normalize the weights of the selected features, and perform element-wise product of the original feature data and the weights.

## Data Availability

Data collection and sharing for this project is funded by the Alzheimer’s Disease Neuroimaging Initiative (ADNI) (National Institutes of Health, USA).
